# Dissipation, persistence, and risk assessment of fluxapyroxad and penthiopyrad residues in perilla leaf (*Perilla frutescens var*. *japonica Hara*)

**DOI:** 10.1371/journal.pone.0212209

**Published:** 2019-04-09

**Authors:** Hyun H. Noh, Jae Y. Lee, Hyo K. Park, Jung W. Lee, Seung H. Jo, Jun B. Lim, Hyun G. Shin, Hyeyoung Kwon, Kee S. Kyung

**Affiliations:** 1 Department of Agro-food Safety and Crop protection, National Institute of Agricultural Sciences, Rural Development Administration, Wanju, Jeonbuk, Republic of Korea; 2 Residue Research Team, Research Institute, NongHyup Chemical, Okcheon, Republic of Korea; 3 Department of Environmental and Biological Chemistry, College of Agriculture, Life and Environment Science, Chungbuk National University, Cheongju, Chungbuk, Republic of Korea; University of Kentuky, UNITED STATES

## Abstract

The objective of this study was to determine the residual characteristics and to calculate the persistence of the fungicides fluxapyroxad (15.3% suspension concentrate) and penthiopyrad (20% emulsifiable concentrate) on the leaves of greenhouse-cultivated perilla (*Perilla frutescens var*. *japonica Hara*). Fluxapyroxad was diluted 2,000-fold and penthiopyrad was diluted 4,000-fold. Each solution was sprayed 3 times onto crops at 7-d intervals before harvest. Leaf samples were collected at 3 h (0 d), 1, 3, 5 and 7 d after the third and final treatment. The recovery ranges of fluxapyroxad and penthiopyrad and their metabolites were 74.2%–104.1%. Pesticide residue analyses indicated that fluxapyroxad and penthiopyrad residues in perilla leaves dissipated over time. The persistence of fluxapyroxad and penthiopyrad residues 7 d after the final spray were 50.0% ± 4.9% and 44.2% ± 2.8% of those measured 3 h (0 d) after the final spray, respectively. The percent acceptable daily intake (%ADI)—which was assessed according to the daily food intake by Koreans according to age—was < 7.3%. Therefore, it was determined that the health risk was low. The perception that residual pesticides are present in large amounts in perilla leaf has led to consumer concern. However, in this study, the amounts of pesticide in perilla leaf decreased over time. Although it has been hypothesized that the risk of pesticide intake would be higher in younger children, the results actually suggest the opposite. Therefore, the pesticides in question are considered to be safe for use on perilla leaves.

## Introduction

Pesticides help to improve and maintain crop quality, and are widely used globally [1] to protect crops against harmful biotic factors such as insect predators, pathogens, and weeds [2,3]. Sprayed pesticides dissipate and are degraded by enzymes, hydrolysis, and exposure to light. However, pesticide residues may persist in and on the surfaces of crops, and can therefore be consumed by humans [4]. While food processing can significantly reduce the quantity of residual pesticide in crops, many fruits and vegetables are directly consumed without processing, and thus the risk of ingesting residual pesticides is very high [5]. Pesticide residue levels in foods are of great concern because these substances have varying degrees of toxicity [6,7]. Moreover, sprayed pesticides may disperse in the atmosphere, run off into nearby waters, and leach into soils. Consequently, pesticides contribute to environmental pollution [1].

Leafy vegetables with high leaf area-to-leaf weight ratios [8] tend to accumulate higher levels of pesticide residue than fruits or non-leafy vegetables. In the latter cases, pesticide residues are diluted by rapid and substantial plant growth. However, the leaves of perilla—the test crop of the present study—accumulate more residual pesticides than many other vegetables because the hairs on the leaves cause the chemicals to be strongly adhered to them. Moreover, perilla does not significantly increase in weight as it grows. For these reasons, perilla is a suitable model in which to test the accumulation of pesticide residues.

The carboxamide fungicides fluxapyroxad (3-(difluoromethyl)-1-methyl-*N*-(3′,4′,5′-trifluorobiphenyl-2-yl)pyrazole-4-carboxamide) and penthiopyrad ((*RS*)-*N*-[2-(1,3-dimethylbutyl)-3-thienyl]-1-methyl-3-(trifluoromethyl)pyrazole-4-carboxamide) are both succinate dehydrogenase inhibitors [9–11]. These agents do not reduce pathogen cross-resistance to benzimidazoles, dicarboximides, anilinopyrimidines, or strobilurins [12]. They are widely used in plant protection. As is the case with other systemic pesticides with long half-lives, fluxapyroxad is readily leached into groundwater. When it is co-applied with pyraclostrobin, the mixture becomes even more susceptible to leaching than fluxapyroxad alone [13]. Furthermore, penthiopyrad is not photodegradable [12].

According to the Codex and Ministry Food and Drug Safety (MFDS) of Korea [14], the parent fluxapyroxad alone is considered when determining its residue levels in foods. However, the Rural Development Administration (RDA) of Korea states that the residual fluxapyroxad level in foods is actually the sum of the parent compound and its metabolites M700F002 and M700F048 [15]. Chen et al. [16] reported that the major plant metabolites of fluxapyroxad are M700F002, M700F008, and M700F048, while the Joint FAO/WHO Meeting on Pesticide Residues (JMPR) [17] stated that only M700F002 and M700F048 are the main fluxapyroxad metabolites. Both the Codex and MFDS (Korea) consider only the parent penthiopyrad when assessing its food residue levels [14]. When the RDA of Korea conducts field trials to analyze residual penthiopyrad levels in crops, they calculate the sum of the parent compound and its metabolites 753-A-OH and PAM [15]. When penthiopyrad foliar spray was applied to cabbage, and the leaves were analyzed for pesticide residues, the major residue was shown to consist of the parent penthiopyrad. PAM was detected in the range of 10%–11% and was regarded as the major penthiopyrad metabolite [17].

Thus, the objectives of this study were i) to determine the residual characteristics of fluxapyroxad and penthiopyrad and their metabolites in perilla leaves over time (sampling dates), ii) to calculate the persistence of the residues, and iii) to assess the risks of the pesticide residues to human health on the basis of measured amounts of residue. The methodology used in the present study could provide a scientific basis for assessing the safety of pesticide residues in leafy vegetables.

## Materials and methods

### Field trials

Fluxapyroxad and penthiopyrad were tested in this study because they are translocated in plants [18,19]. The test crop—perilla leaf—was cultivated under greenhouse conditions. The treatment plots were 10 m^2^ in area and were arranged in triplicate. To minimize spray overlap, buffer zones (1 m) were set up between plots. The commercial products investigated were fluxapyroxad 15.3% suspension concentrate (SC) and penthiopyrad 20% emulsifiable concentrate (EC), which were diluted 2,000-fold and 4,000-fold, respectively. The products were sprayed onto perilla leaves a total of 3 times at 7-d intervals prior to harvest on the last spraying day. Perilla leaf samples were collected 3 h (0 d), 1, 3, 5, and 7 d after the third and final spray [20], immediately, homogenized on dry ice using a blender, were subsequently packed in polyethylene bags, and stored at −20°C until pesticide residue analyses [20].

### Recovery test

The recovery test method was validated for fluxapyroxad by fortifying 5 g of perilla leaf with 0.1 mg kg^−1^ and 0.5 mg kg^−1^. In addition, 5 g of perilla leaf was fortified with 0.5 mg kg^−1^ and 2.5 mg kg^−1^ to validate the analytical methods for the metabolites of fluxapyroxad (i.e., M700F002 and M700F048). The recovery test method was also validated for penthiopyrad and its metabolites 753-A-OH and PAM by fortifying 10 g of perilla leaf with 0.1 mg kg^−1^ and 0.5 mg kg^−1^. Samples were prepared in triplicate for each pesticide tested. Finally, recovery for the test pesticides and their metabolites were fortified at 10-fold and 50-fold of the LOQ (limit of quantitation) for each compound. As the test methods were successfully validated, they were used in the subsequent pesticide analyses of perilla leaf samples.

### Storage stability

The Health & Safety Executive (HSE) [21] reported that a storage stability test should be performed to ensure that the pesticide in the sample does not substantially degrade upon exposure to environmental factors such as temperature, humidity, and light. Thus, the stability of the pesticides in the samples during freezer storage was evaluated. One milliliter of a 2.5 mg L^−1^ fluxapyroxad standard was mixed with 5 g of untreated perilla leaf, resulting in a final concentration of 0.5 mg kg^−1^. Three replicates of 10 g of untreated perilla leaf were fortified with 1 mL of 12.5 mg L^−1^ M700F002 and 1 mL of 12.5 mg L^−1^ M700F048, bringing the final concentrations to 2.5 mg kg^−1^. For penthiopyrad and its metabolites, each of the three replicated samples of untreated perilla leaf (10 g) was fortified with 1 mL of 5 mg L^−1^ of the test substances. All samples were stored at −20°C until analysis. Stabilities were validated by calculating the percent recovery.

### Sample preparation

#### Fluxapyroxad and its metabolites

Five grams of perilla leaf was weighed in a tall beaker (300 mL) and 50 mL acetone was added. The mixture was homogenized in a blender at 10,000 rpm for 3 min. The homogenate was then vacuum-filtered using filter paper (Whatman No. 2 (8 μm), USA) and Celite 545 (Merck, USA). The flask and filter cake were rinsed with 50 mL acetone and the rinsate was combined with the filtrate.

The filtrate was quantitatively transferred to a 1 L separatory funnel, to which 400 mL of distilled water, 100 mL of saturated NaCl solution, and 50 mL of dichloromethane were added in sequence. For metabolite determination, 50 mL of ethyl acetate was substituted for the dichloromethane. The mixture was vigorously agitated on a shaker at 250 rpm for 10 min and allowed to stand until two distinct layers formed. The organic solvent layer was filtered through 20 g of anhydrous Na_2_SO_4_ and collected in a 250 mL distillation flask. The partition process was repeated using another 50 mL of dichloromethane (or 50 mL of ethyl acetate in the case of the metabolites), and the organic layers were pooled in the distillation flask. The organic solvent extract was evaporated to dryness at 35°C on a rotary vacuum evaporator (Buchi, R-205V, Gemany). The residues were redissolved in 5 mL of acetonitrile, filtered with a 0.2 μm syringe filter, and analyzed by liquid chromatography-tandem mass spectrometry (LC-MS/MS) (Table 1).

**Table 1:** Instrumentation and settings used in the analysis of fluxapyroxad and its metabolites M700F002 and M700F048 in perilla leaf.

Instrument	Acquity UHPLC H class system, Waters Corporation, Milford, MA, USA					
Data processing	MassLynx v. 4.1					
Detector	Triple-quadruple spectrometer, Xevo TQD, Waters Corporation, Milford, MA, USA					
Column	Phenomenex Kinetex 2.6 μm C18 100A (Phenomenex, Torrance, CA, USA), (150 mm L. × 2.1 mm I.D.)					
Mobile phase	Acetonitrile: 0.1% formic acid (80:20, v/v) for fluxapyroxad Acetonitrile: 0.1% formic acid (90:10, v/v) for metabolites					
Flow rate	0.3 mL min^−1^					
Injection volume	5.0 μL for fluxapyroxad, 10.0 μL for metabolites					
Ionization source	Electrospray ionization (ESI)					
Polarity	Positive for fluxapyroxad, negative for metabolites					
Source Temp.	120°C					
Capillary voltage	3.0 kV					
Cone gas flow	50 L h^−1^					
Desolvation gas	Temperature: 350°C; Flow rate: 600 L h^−1^					
MRM conditions were as follows:						
Compound	Precursor (m/z)	CV^a^ (V)	Quantification (m/z)	CE^b^ (V)	Confirmation (m/z)	CE (V)
Fluxapyroxad	382.26	34	342.24	20	362.24	20
M700F002	161.32	20	141.25	9	117.16	9
M700F048	528.31	48	346.15	19	366.14	15

^a^Cone voltage

^b^Collision energy

#### Penthiopyrad and its metabolites

Ten grams of perilla leaf was weighed in a tall beaker (300 mL) and 50 mL of acetone was added. The mixture was homogenized in a blender at 10,000 rpm for 2 min. The homogenate was then vacuum-filtered using filter paper (8 μm) and Celite 545. The flask and filter cake were rinsed with 50 mL of acetone and the rinsate was combined with the filtrate.

The filtrate was quantitatively transferred to a 1 L separatory funnel, to which 400 mL of distilled water, 100 mL of saturated NaCl solution, and 50 mL of ethyl acetate were added in sequence. The mixture was vigorously agitated on a funnel shaker at 250 rpm for 5 min and allowed to stand until two distinct layers formed. The organic solvent layer was filtered through 20 g of anhydrous Na_2_SO_4_ and collected in a 250 mL distillation flask. The partition process was repeated using another 50 mL of ethyl acetate and the organic layers were pooled in the distillation flask. The organic solvent extract was evaporated to dryness at 35°C on a rotary vacuum evaporator. The residue was re-dissolved in 5 mL of dichloromethane and subjected to Florisil column chromatography for sample purification.

A chromatographic glass column (35 cm L × 1.1 cm I.D.) was dry-packed with 5 g of activated Florisil at 130°C over a 5 h period and topped with approximately 2 cm (~2 g) of anhydrous Na_2_SO_4_. Fifty milliliters of dichloromethane were added to the column. When the solvent level reached the top of the anhydrous Na_2_SO_4_, 5 mL of redissolved sample was applied to the column followed by another 5 mL of dichloromethane to wash the sample flask. The column was washed with 50 mL of dichloromethane and the solvent discarded. PAM was eluted with a 50 mL mixture of dichloromethane:acetone (90:10, v/v). Penthiopyrad and 753-A-OH were continually eluted with a 50 mL mixture of dichloromethane:acetone (50:50, v/v). The fractions were collected and concentrated to dryness at 35°C on a vacuum rotary evaporator. The residue was redissolved in 2 mL of acetone for determination using a gas chromatography-nitrogen phosphorus detector (GC-NPD) (Table 2).

**Table 2:** Instrumentation and settings used in the analysis of penthiopyrad and its metabolites 753-A-OH and PAM in perilla leaf.

Instrument	7890A gas chromatograph, Agilent Technologies, Santa Clara, CA, USA			
Column	DB-5, 30 m L. matograph, Agilent Teμm film thickness)			
Detector	Nitrogen phosphorus detector (NPD)			
Temperature	Penthiopyrad and 753-A-OH	Increase rate (°C min^-1^)	Temperature	Hold time (min)
			100	1
		20	150	1
		20	230	1
		10	250	1
		2	260	2
	PAM	Increase rate (°C min^-1^)	Temperature	Hold time (min)
			100	1
		20	150	1
		Increase rate (°C min^-1^)	Temperature	Hold time (min)
Gas low rate	Carrier gas (N2)	1.0 mL min^−1^		
	Make up gas (N2)	5.0 mL min^−1^		
	H2	3.0 mL min^−1^		
	Air	120 mL min^−1^		
Injection volume	1 μL			
Split mode	Splitless			

### Total amount of pesticide

The total amount of pesticide was the sum of the concentrations of the parent compound and the metabolites divided by the ratio of the molecular weight of the parent compound and the metabolites (Eqs 1 and 2) (FAO, 2016).

$$Total\;\left(mg\;kg^{‐1}\right)=Parent\;compound\;\left(mg\;kg^{‐1}\right)+\left[Metabolite\;\left(mg\;kg^{‐1}\right)\times Conversion\;factor\right]$$ (1)

Conversionfactor=Molecularweightofparent/Molecularweightofmetabolite (2)

### Risk assessment

The risks to human health (percent acceptable daily intake; %ADI) of the test pesticides ingested with perilla leaf were calculated by the ratio of estimated daily intake (EDI) to the acceptable daily intake (ADI). The high residue (HR) and daily food intake of perilla leaf by Koreans according to age were multiplied to obtain the estimated daily intakes (EDIs). The food daily intake of perilla leaf by age for Koreans was derived from the Korea Health Industry Development Institute [22]. The average body weights of Koreans by age were acquired from the Korea Centers for Disease Control and Prevention [23]. The EDIs were calculated using average body weights by age. Eqs 3, 4 and 5 (below) were used for the risk assessment [24]. The ADIs were determined using data from the Pesticide and Veterinary Drugs Information database (http://www.foodsafetykorea.go.kr/residue/main.do) provided by the Korean MFDS. In addition, TMDIs (Theoretical Maximum Daily Intakes) of test pesticides for other crops were calculated using Eq (6) and maximum residue limits (MRLs) were used from the Korean MFDS. TMDIs were calculated using the average body weight (57.8 kg) of adults (over 19 y) and MRLs in Korea.

$$ADI\;\left(mg\;kg\;person^{‑1}\right)=ADI\;\left(mg\;kg^{‑1}\cdot body\;weight\;day^{‑1}\right)\;of\;test\;pesticide\times average\;body\;weight\;by\;age$$ (3)

$$EDI\;\left(mg\;kg\;person^{-1}\right)=amount\;of\;test\;pesticide\;(mg\;kg^{-1})\times food\;daily\;intake\;\left(g\right)$$ (4)

%ADI=EDI/ADI×100 (5)

TMDI(%)=∑%ADIofregisteredallcrops (6)

## Results

### Recovery test

Table 3 shows that the recoveries of fluxapyroxad and its metabolites M700F002 and M700F048 from perilla leaf were 90.4%–98.7%, 74.2%–81.7%, and 77.4%–84.5%, respectively. The recoveries of penthiopyrad and its metabolites 753-A-OH and PAM from perilla leaf were 89.5%–102.1%, 81.1%–104.1%, and 78.4%–81.4%, respectively. These results were similar to those reported in other studies. Abad-Fuentes et al. [25] indicated that the recoveries of fluxapyroxad and penthiopyrad from apple, strawberry, tomato, and spinach ranged from 70% to 120%. Five pyrazole pesticides were analyzed in rice, wheat, cucumber, tomato, lettuce, apple, and grape by Dong et al. [26] using the “Quick-Easy-Cheap-Effective-Rugged-Safe” (QuEChERS) method and ultra-high-performance liquid chromatography/tandem mass spectrometry (UHPLC-MS/MS). The recovery rates for the five pyrazole fungicides were 75.9%–108.0%. This experiment demonstrates the suitability of the pesticide residue analytical techniques used in the present study.

**Table 3:** Recoveries of the test pesticides and their metabolites in perilla leaf.

Pesticide	Fortification (mg kg^−1^)	Recovery (%)					CV^b^ (%)
		Rep. 1	Rep. 2	Rep. 3	Mean	SD^a^	
Fluxapyroxad	0.1	90.4	98.7	94.7	94.6	4.2	4.4
	0.5	96.7	93.3	94.3	94.8	1.8	1.9
M700F002	0.5	74.2	81.7	78.1	77.9	3.7	4.8
	2.5	81.2	81	81.3	81.2	0.2	0.2
M700F048	0.5	78.1	77.4	75.9	77.8	1.1	1.4
	2.5	83.3	83.9	84.5	83.9	0.6	0.7
Penthiopyrad	0.1	102.1	98.4	94.9	98.5	3.6	3.7
	0.5	95.2	89.5	94.9	93.2	3.2	3.5
753-A-OH	0.1	104.1	91.6	95.1	96.9	6.5	6.7
	0.5	84.4	81.1	88.8	84.8	3.8	4.5
PAM	0.1	78.4	79.9	81.4	79.9	1.5	1.9
	0.5	80.2	79.6	80.4	80.1	0.4	0.5

^a^Standard deviation

^b^Coefficient of variation

### Storage stability

The storage stability tests for fluxapyroxad, penthiopyrad, and their metabolites in perilla leaf generated recovery rates of 80.5%–98.8% and 77.8%–101.5%, respectively (Table 4). Therefore, the pesticides tested in the present study and their metabolites were stable during storage, as the recovery rates ranged from 70% to 120% [20].

**Table 4:** Storage stabilities of the test pesticide and their metabolites in perilla leaf.

Pesticide	Storage period (day)	Fortification (mg kg^−1^)	Recovery (%)					CV^b^ (%)
			Rep. 1	Rep. 2	Rep. 3	Mean	SD^a^	
Fluxapyroxad	69	0.5	94.2	98.8	91.9	95.0	3.5	3.7
M700F002	71	2.5	85.8	83.2	83.0	84.0	1.6	1.9
M700F048	71	2.5	82.1	84.3	80.5	82.3	1.9	2.3
Penthiopyrad	69	0.5	98.7	96.0	101.5	98.7	2.7	2.8
753-A-OH	68	0.5	88.6	91.8	85.1	88.5	3.4	3.8
PAM	60	0.5	77.8	79.5	79.5	78.9	1.0	1.2

^a^Standard deviation

^b^Coefficient of variation

### Residual pesticide

The average concentration of fluxapyroxad in perilla leaf on the final day of spraying (3 h) was 20.88 ± 1.58 mg kg^−1^ (Table 5). Seven days after the final spray, the average fluxapyroxad concentration in perilla leaf was 10.45 ± 1.11 mg kg^−1^. Therefore, the fluxapyroxad content in perilla leaf decreased over time. The amount of fluxapyroxad metabolites were less than the LOQ. The JMPR (2013) reported that while the major fluxapyroxad metabolites were M700F002 and M700F048, they were detected at levels below the LOQ. The average penthiopyrad concentration in perilla leaf on the final day of spraying (day 0) was 11.19 ± 0.67 mg kg^−1^ (Table 6). Seven days after the final spray, the average penthiopyrad concentration in perilla leaf was 4.79 ± 0.12 mg kg^−1^. There was almost no change in the concentration of 753-A-OH in perilla leaf from days 0 to 7 (Table 7). However, JMPR (2013) reported that the residual concentration of PAM in cabbage sprayed with penthiopyrad steadily increased from day 0 (0.15 ± 0.01 mg kg^−1^) to day 7 (0.30 ± 0.02 mg kg^−1^). According to FAO (2016), the total penthiopyrad concentrations in perilla leaf were 11.53 ± 0.70 mg kg^−1^ on day 0 and 5.08 ± 0.10 mg kg^−1^ on day 7. Therefore, in the present study, as well as in previous studies, the residue levels of penthiopyrad decreased over time. The total pesticide concentrations were used to calculate persistence and %ADI. No MRLs have yet been established for fluxapyroxad or penthiopyrad in perilla leaf.

**Table 5:** Residues of fluxapyroxad and its metabolites in perilla leaf.

Pesticide	Day after last application (day)	Mean residue ± SD^a^ (mg kg^−1^)	Coefficient of variation (%)
Fluxapyroxad	0	20.88 ± 1.58	7.55
	1	18.82 ± 0.74	3.94
	3	15.15 ± 1.44	9.48
	5	13.11 ± 1.56	11.92
	7	10.45 ± 1.11	10.59
M700F002	0	< 0.05^b^	-
	1	< 0.05	-
	3	< 0.05	-
	5	< 0.05	-
	7	< 0.05	-
M700F048	0	< 0.05	-
	1	< 0.05	-
	3	< 0.05	-
	5	< 0.05	-
	7	< 0.05	-

^a^Standard deviation

^b^Less than the LOQ

**Table 6:** Residues of penthiopyrad and its metabolites in perilla leaf.

Pesticide	Day after last application (day)	Mean residue ± SD^a^ (mg kg^−1^)	Coefficient of variation (%)
Penthiopyrad	0	11.19 ± 0.67	6.02
	1	9.16 ± 0.32	3.46
	3	7.17 ± 0.47	6.62
	5	5.51 ± 0.34	6.08
	7	4.79 ± 0.12	2.59
753-A-OH	0	0.05 ± 0.01	10.26
	1	0.05 ± 0.00	9.52
	3	0.04 ± 0.00	4.29
	5	0.04 ± 0.00	10.64
	7	0.04 ± 0.00	4.95
PAM	0	0.15 ± 0.01	8.91
	1	0.19 ± 0.01	4.11
	3	0.24 ± 0.01	4.65
	5	0.26 ± 0.00	1.67
	7	0.30 ± 0.02	6.89
Total Penthiopyrad	0	11.53 ± 0.70	6.03
	1	9.56 ± 0.30	3.18
	3	7.66 ± 0.47	6.15
	5	6.02 ± 0.35	5.74
	7	5.08 ± 0.10	1.87

^a^Standard deviation

**Table 7:** Time course of persistence of fluxapyroxad and penthiopyrad based on total residue in perilla leaf.

Pesticide	Day since last application (day)	Mean persistence ± SD^a^ (%)	Coefficient of variation (%)
Fluxapyroxad	1	90.1 ± 4.6	5.1
	3	72.6 ± 11.2	15.5
	5	62.8 ± 6.6	10.5
	7	50.0 ± 4.9	9.8
Penthiopyrad	1	83.3 ± 7.9	9.4
	3	66.6 ± 5.1	7.7
	5	52.2 ± 2.6	4.9
	7	44.2 ± 2.8	6.4

^a^Standard deviation

### Pesticide residue persistence

Pesticide residue persistence in perilla leaf was determined by collecting and analyzing treated leaf samples 1, 3, 5, and 7 d after the final spray. Fluxapyroxad persistence was calculated according to its concentrations in perilla leaf from day 0 (Table 7). Relative to day 0 levels, fluxapyroxad persistence levels on days 1 and 7 were 90.1% ± 4.6% and 50.0% ± 4.9%, respectively. Penthiopyrad persistence was calculated using the same method used for fluxapyroxad and was found to be 83.3% ± 7.9% at day 1 and 44.2% ± 2.8% by day 7. Since both test pesticides are pyrazoles, they exhibited similar persistence patterns. Their residues persisted for longer periods of time in perilla leaf than they did in crops like cucumber and squash, whose fruit rapidly increases in weight as they grow [27].

### Risk assessment

The human health risks associated with the ingestion of the test pesticides via the crops treated with them were assessed by comparing EDIs and ADIs at an HR level. The %ADIs of fluxapyroxad and penthiopyrad in perilla leaf in Koreans by age are presented in Table 8. The %ADIs tended to increase with age, where the rates of increase in daily food intake are greater than those associated with body weight. From ages 12 to 18 y, the %ADIs of fluxapyroxad and penthiopyrad were 3.7% and 0.5%, respectively. From ages 19 to 29 y, they were 6.4% and 0.9%, respectively. In the latter case, body weight did not significantly increase, while daily food intake doubled relative to the former. The %ADI of the 50–64 y age group was the highest of all measured (7.2% for fluxapyroxad and 1.0% for penthiopyrad, respectively). It is the relative difference in ADI rather than pesticide concentration, body weight, or daily food intake that actually influences %ADI calculations. If the ADI is high, then the %ADI will be low irrespective of pesticide concentration or daily food intake. Additionally, test pesticides without MRLs could not be compared in terms of residual amounts. However, when compared with ADI, the health risk was found to be low. In addition, TMDIs have been calculated to assess safety for fluxapyroxad and penthiopyrad in registered all crops in Korea (Tables 9 and 10). The TMDIs of fluxapyroxad and penthiopyrad were 35.64 and 9.52%, respectively. Therefore, the risk to human health for the test pesticides in registered crops in Korea is considered to be low. Also, considering that the Korean government ensures that TMDIs do not exceed 80% of the ADI for a given pesticide, the test pesticides are considered safe.

**Table 8:** %ADI for the risk assessment of fluxapyroxad and penthiopyrad in perilla leaf in Koreans by age.

Pesticide	Age	Food daily intake (g)	Residue (mg kg^−1^)	EDI^a^ (mg kg^−1^ bw^b^ day^−1^)	Body weight (kg)	ADI^c^ (mg person^−1^ day^−1^)	%ADI
Fluxa-pyroxad	1–2	0.2	20.88	0.0038	12.2	0.256	1.5
	3–5	0.2	20.88	0.0042	17.2	0.361	1.2
	6–11	0.9	20.88	0.0192	30.0	0.630	3.0
	12–18	1.9	20.88	0.0405	52.4	1.100	3.7
	19–29	3.9	20.88	0.0818	61.1	1.282	6.4
	30–49	4.1	20.88	0.0850	58.9	1.237	6.9
	50–64	4.1	20.88	0.0858	56.4	1.184	7.2
	≥ 65	3.3	20.88	0.0693	54.7	1.149	6.0
Penthio-pyrad	1–2	0.2	11.53	0.0021	12.2	0.988	0.2
	3–5	0.2	11.53	0.0023	17.2	1.393	0.2
	6–11	0.9	11.53	0.0106	30.0	2.430	0.4
	12–18	1.9	11.53	0.0224	52.4	4.243	0.5
	19–29	3.9	11.53	0.0452	61.1	4.945	0.9
	30–49	4.1	11.53	0.0469	58.9	4.771	1.0
	50–64	4.1	11.53	0.0474	56.4	4.568	1.0
	≥ 65	3.3	11.53	0.0383	54.7	4.431	0.9

^a^Estimated daily intake

^b^Body weight

^c^Acceptable daily intake

**Table 9:** TMDIs for the risk assessment of fluxapyroxad for registered crops in Korea.

Crop	Food daily intake (g)	MRL^a^ (mg kg^−1^)	EDI^b^ (mg kg^−1^ bw^c^ day^−1^)	ADI^d^ (mg person^−1^ day^−1^)	%ADI	TMDI^e^ (%)
Eggplants	3.00	0.5	0.0015	1.214	0.12	35.64
Persimmons	20.52	0.3	0.0062	1.214	0.51	
Citrus fruits	20.06	1.0	0.0201	1.214	1.65	
Potatoes	20.72	0.02	0.0004	1.214	0.03	
Ginseng (dried)	0.39	0.2	0.0001	1.214	0.01	
Nuts	7.85	0.05	0.0004	1.214	0.03	
Peppers	27.37	1.0	0.0274	1.214	2.25	
Soy beans	3.94	0.15	0.0006	1.214	0.05	
Beans	35.81	0.3	0.0107	1.214	0.89	
Perilla leaves	3.43	20.88	0.0716	1.214	5.90	
Strawberries	3.65	2.0	0.0073	1.214	0.60	
Peanuts	1.06	0.01	0.0000	1.214	0.00	
Garlic	4.76	0.05	0.0002	1.214	0.02	
Mangos	0.30	0.5	0.0002	1.214	0.01	
Korean plums	0.57	1.0	0.0006	1.214	0.05	
Melons	1.55	0.5	0.0008	1.214	0.06	
Wheat	0.00	0.3	0.0000	1.214	0.00	
Bananas	10.77	3.0	0.0323	1.214	2.66	
Pears	14.88	0.8	0.0119	1.214	0.98	
Korean cabbage	8.52	0.05	0.0004	1.214	0.04	
Barley	6.28	2.0	0.0126	1.214	1.03	
Peaches	10.92	0.3	0.0033	1.214	0.27	
Chives	2.91	5.0	0.0146	1.214	1.20	
Blueberries	0.78	7.0	0.0055	1.214	0.45	
Apples	53.96	0.5	0.0270	1.214	2.22	
Sugar beets	0.03	0.1	0.0000	1.214	0.00	
Celery	0.15	10	0.0015	1.214	0.12	
Watermelons	16.55	0.1	0.0017	1.214	0.14	
Ginseng	0.39	0.05	0.0000	1.214	0.00	
Sorghum	0.65	0.8	0.0005	1.214	0.04	
Rice	151.72	0.05	0.0076	1.214	0.62	
Cabbage	11.01	3.0	0.0330	1.214	2.72	
Lettuce	1.50	15.0	0.0225	1.214	1.85	
Onions	28.53	0.05	0.0014	1.214	0.12	
Cucumbers	16.37	0.2	0.0033	1.214	0.27	
Corn	3.62	0.15	0.0005	1.214	0.04	
Peas	0.33	0.4	0.0001	1.214	0.01	
Rape seed	0.60	0.8	0.0005	1.214	0.04	
Plums	2.63	1.5	0.0039	1.214	0.33	
Sesame seeds	0.63	0.3	0.0002	1.214	0.02	
Korean melons	11.07	0.3	0.0033	1.214	0.27	
Chwinamul	1.58	10.0	0.0158	1.214	1.30	
Tomatoes	18.28	1.0	0.0183	1.214	1.51	
Welsh onions	12.93	2.0	0.0259	1.214	2.13	
Grapes	15.09	2.0	0.0302	1.214	2.49	
Green garlic	0.75	0.5	0.0004	1.214	0.03	
Sweet peppers	0.88	1.0	0.0009	1.214	0.07	
Sunflower seeds	0.06	0.2	0.0000	1.214	0.00	
Squash	11.26	0.5	0.0056	1.214	0.46	

^a^Maximum residue limit

^b^Estimated daily intake

^c^Body weight

^d^Acceptable daily intake

^e^Theoretical maximum daily intake

**Table 10:** TMDIs for the risk assessment of penthiopyrad for registered crops in Korea.

Crop	Food daily intake (g)	MRL^a^ (mg kg^−1^)	EDI^b^ (mg kg^−1^ bw^c^ day^−1^)	ADI^d^ (mg person^−1^ day^−1^)	%ADI	TMDI^e^ (%)
Eggplants	3.00	2	0.0060	4.682	0.13	9.52
Persimmons	20.52	0.7	0.0144	4.682	0.31	
Mandarins	20.06	0.7	0.0140	4.682	0.30	
Ginseng (dried)	0.39	0.2	0.0001	4.682	0.00	
Nuts	7.85	0.05	0.0004	4.682	0.01	
Peppers	27.37	3	0.0821	4.682	1.75	
Mung beans	0.12	0.05	0.0000	4.682	0.00	
Perilla leaves	3.43	11.53	0.0395	4.682	0.84	
Strawberries	3.65	1	0.0037	4.682	0.08	
Peanuts	1.06	0.04	0.0000	4.682	0.00	
Garlic	4.76	0.05	0.0002	4.682	0.01	
Korean plums	0.57	1	0.0006	4.682	0.01	
Pears	14.88	0.5	0.0074	4.682	0.16	
Peaches	10.92	0.2	0.0022	4.682	0.05	
Chives	2.91	2	0.0058	4.682	0.12	
Apples	53.96	0.2	0.0108	4.682	0.23	
Lettuces	7.64	20	0.1528	4.682	3.26	
Watermelons	16.55	0.1	0.0017	4.682	0.04	
Ginseng	0.39	0.1	0.0000	4.682	0.00	
Onions	28.53	0.7	0.0200	4.682	0.43	
Mulberries	0.18	2	0.0004	4.682	0.01	
Cucumbers	16.37	0.5	0.0082	4.682	0.17	
Plums	2.63	0.2	0.0005	4.682	0.01	
Korean melons	11.07	0.5	0.0055	4.682	0.12	
Tomatoes	18.28	2	0.0366	4.682	0.78	
Grapes	15.09	2	0.0302	4.682	0.64	
Green garlic	0.75	0.05	0.0000	4.682	0.00	
Sweet peppers	0.88	3	0.0026	4.682	0.06	

^a^Maximum residue limit

^b^Estimated daily intake

^c^Body weight

^d^Acceptable daily intake

^e^Theoretical maximum daily intake

## Discussion

Pesticide concentrations in perilla leaves were likely affected by various factors, including the physicochemical properties of the pesticide [28], crop morphology [8], and environmental factors such as weather and cultivation conditions [29]. The pesticide concentrations in perilla leaf were high because they are absorbed systemically and translocated in the plant [18,19]. Paterson et al. [30] reported that pesticide translocation and systemic properties significantly influence crop residue levels, while Park et al. [31] stated that systemic pesticides are more persistent than non-systemic ones. In another study, the systemic insecticide imidacloprid was sprayed onto eggplant after bloom and before fruit set. The plants were then analyzed for imidacloprid residues up until harvest, and imidacloprid residues were detected in the fruit following the foliar application of the pesticide because it was translocated from the leaves [32].

Crop morphology also determines residual pesticide concentrations. Residue levels tend to be relatively higher in crops with hairy shoot surfaces [33]. As foliar sprays deposit high concentrations of pesticide on hairy leafy crops like perilla, residual pesticide concentrations in perilla leaf were higher than those in crops with relatively smooth leaves. Moreover, perilla has a comparatively large leaf surface area [8].

Pesticide residue concentrations in fruiting vegetables such as cucumber and squash quickly decrease over time because the pesticides are diluted as the crops rapidly grow [34]. In contrast, perilla leaf grows slowly and does not significantly increase in weight with time. Therefore, there was no dilution effect of the pesticide in perilla leaves and its residue levels remained relatively high. However, it is important to note that the perilla plants used in the present study were cultivated under controlled greenhouse conditions. Consequently, the dilution and rinsing effects of rainfall did not occur, and comparatively larger amounts of the pesticides could be absorbed and translocated by the leaves [35].

Individuals in the youngest age group would have the lowest body weights. Therefore, it is expected that the youngest individuals would experience the highest relative health risk from ingesting perilla leaf containing residues of the test pesticides. Nevertheless, their daily fresh vegetable intake is comparatively low. As fresh vegetable consumption levels increase with age, so does the risk of dietary pesticide exposure. However, the health risks are by no means equally high in all Koreans. Chun and Kang [35] reported that when %ADI is < 10%, the relative risk is low and no further analysis is required. Even when 10 ≤ %ADI ≤ 30, the pesticide residue concentration poses no significant health risk. In the present study, the maximum %ADI was 7.2% and TMDIs of fluxapyroxad and penthiopyrad were 35.64 and 9.52%, respectively. Therefore, the fluxapyroxad and penthiopyrad residue concentrations in perilla leaves pose no significant health risks to Koreans.

## Conclusions

The aim of the present study was to determine the dissipation characteristics of two pyrazole fungicides sprayed at time intervals onto perilla leaves under greenhouse conditions. Another objective was to assess the health risks that ingesting perilla leaves treated with these fungicides might pose to Koreans of various ages. The test results of pesticide residues in perilla leaves indicates that the substances dissipate over time, although not significantly. The health risk assessment demonstrated that the residues present in perilla leaves do not pose a health threat to Koreans of any age group.
